# PPIGCF: A Protein–Protein Interaction-Based Gene Correlation Filter for Optimal Gene Selection

**DOI:** 10.3390/genes14051063

**Published:** 2023-05-10

**Authors:** Soumen Kumar Pati, Manan Kumar Gupta, Ayan Banerjee, Saurav Mallik, Zhongming Zhao

**Affiliations:** 1 Department of Bioinformatics, Maulana Abul Kalam Azad University of Technology, Haringhata 741249, West Bengal, India; 2 Department of Computer Science and Engineering, Jalpaiguri Govt. Engineering College, Jalpaiguri 735102, West Bengal, India; 3 Center for Precision Health, School of Biomedical Informatics, The University of Texas Health Science Center at Houston, Houston, TX 77030, USA; smallik@arizona.edu; 4 Department of Environmental Health, Harvard T H Chan School of Public Health, Boston, MA 02115, USA; 5 Department of Pharmacology & Toxicology, University of Arizona, Tucson, AZ 85721, USA; 6 Human Genetics Center, School of Public Health, The University of Texas Health Science Center at Houston, Houston, TX 77030, USA

**Keywords:** dimension reduction, protein–protein interaction, gene ontology, Pearson’s correlation, information content

## Abstract

Biological data at the omics level are highly complex, requiring powerful computational approaches to identifying significant intrinsic characteristics to further search for informative markers involved in the studied phenotype. In this paper, we propose a novel dimension reduction technique, protein–protein interaction-based gene correlation filtration (PPIGCF), which builds on gene ontology (GO) and protein–protein interaction (PPI) structures to analyze microarray gene expression data. PPIGCF first extracts the gene symbols with their expression from the experimental dataset, and then, classifies them based on GO biological process (BP) and cellular component (CC) annotations. Every classification group inherits all the information on its CCs, corresponding to the BPs, to establish a PPI network. Then, the gene correlation filter (regarding gene rank and the proposed correlation coefficient) is computed on every network and eradicates a few weakly correlated genes connected with their corresponding networks. PPIGCF finds the information content (IC) of the other genes related to the PPI network and takes only the genes with the highest IC values. The satisfactory results of PPIGCF are used to prioritize significant genes. We performed a comparison with current methods to demonstrate our technique’s efficiency. From the experiment, it can be concluded that PPIGCF needs fewer genes to reach reasonable accuracy (~99%) for cancer classification. This paper reduces the computational complexity and enhances the time complexity of biomarker discovery from datasets.

## 1. Introduction

Many methods and tools exist for analyzing omics data [1,2,3,4,5], including those for dealing with mRNA gene expression datasets. In these methods, it is common to consider factors such as the features (e.g., genes or mutations) and the various sample sizes of the collected samples (e.g., disease versus matched standard samples). So, a classification model built on these data will take a more experimental time frame and have increased computational cost. The proposed algorithm intends to provide a novel gene selection technique to reduce computational cost without sacrificing the classification performance. In works by Roweis et al. [6,7], machine learning and statistical methods were used to optimize the number of random variables. Here, the main objective is to recognize the random variables in the mRNA expression dataset. In the biological database, the rows and columns constitute the gene names and samples, respectively. As a large set of genes is not involved in any disease, gene symbols have been taken as a variable. This work aims to reject the genes with less information related to diseases. One of the essential tasks in bioinformatics is to identify novel biomarkers or hub genes for several types of cancer for further clinical treatments. However, identifying those genes is very time-consuming based on the high volume of an omics dataset. So, there is a critical gap between the biomarker discovery field and dimension reduction techniques regarding how to reduce computational complexity and improve time complexity to obtain better gene signatures. In this context, the proposed method identifies a small subset of genes from different cancer data for further experiments to obtain the biological information. A detailed literature review is presented in Section 1.1 to consolidate this claim.

### 1.1. Literature Review

Numerous Computational techniques have been developed to survey the dimensionality reduction of data across several domains. However, dimensionality reduction can be classified into two key sections: factor- or component-based and projection-based techniques. Using a factor-based approach, Cook et al. [8] proposed a novel framework to determine the effect of the accommodation grades of students with disabilities on a reading comprehension assessment based on factor analysis. Later, they modified this method with cartoon formalism and regularization to reduce the dimension based on numerical relativity [9]. In 2005, Chao et al. [10] developed a novel dimension reduction technique for microarray data with locally linear embedding. Teng et al. [11] proposed the same work with local tangent space alignment in the same year.

Similarly, another component-based technique was proposed by Ian T. Jolliffe et al. [12] constructed using Principal Component Analysis (PCA), where an evaluation was made with recent developments. Later, Guo et al. [13] used PCA for L1-regularized feature selection on microarray data [14], which provided a brief overview about the potential of this data mining method. On the other hand, Aapo Hyvarinen and Erkki Oja proposed a component-based technique [15] where Independent Component Analysis (ICA) was presented with several algorithms and applications. In addition, they produced a survey report on the ICA in [16] to draft their findings. Later, Kairov et al. [17] extended this approach to identify the optimal number of components for reproducible transcriptomic microarray data analysis.

Nevertheless, the factor-based technique cannot entirely use dimensionality reduction. This is why a novel paper proposed by Joshua B. Tenenbaum et al. [18] on ISOMAP (Isometric Mapping) gave the authors hands-on experience in a projection-based approach. Later, Sun et al. [19] extended this work and developed UL-ISOMAP, and used it for nonlinear dimensionality reduction. Based on this technique, Canedo et al. [20] proposed a distributed feature selection method for microarray data classification. Yu et al. proposed a dynamic module search of gene co-expression networks and applied it to hepatocellular carcinoma. Laurens van der Maaten and Geoffrey Hinton recently proposed a new approach, namely t-SNE (Stochastic Neighbor Embedding) [21], to reduce the dimension of gene expression data. This work was later transformed into kernel t-SNE [22] and dynamic t-SNE [23]. Based on these techniques, Ebrahimpour et al. [24] reduced the dimension of microarray data with row echelon form to obtain their linear independent features. Later, Leland McInnes et al. [25] presented a novel method, Uniform Manifold Approximation and Projection (UMAP), for dimension reduction based on the theoretical framework of Algebraic topology and Riemannian geometry. In 2019, this method was used for the dimension reduction of a single-cell dataset [26]. Ghosh et al. [27] proposed a recursive memetic algorithm for gene selection in microarray data using this concept. In 2020, Saeid et al. [28] used discrete wavelet transform for data reduction and a genetic algorithm for the feature selection of microarray data. Later, Bhui et al. [29] modified this work with a deep-learning approach to perform data reduction in a single step. The paper [30] addressed a filter-based feature selection method from microarray data. Nouri-Moghaddam et al. [31] proposed a unique technique, a novel bio-inspired hybrid multi-filter wrapper gene selection method, to reduce the dimension of gene expression data.

In recent decades, several metaheuristic feature selection techniques have been developed that can effectively select the best features while minimizing the loss of information [32]. This shows the importance of optimization techniques during the feature selection process. Regarding this process, Kundu et al. [33] proposed an Altruistic Whale Optimization Algorithm for the feature selection of microarray gene expression data. This algorithm is derived from observations of the whale population and assists in the productive spread of applicant arrangements that, with canning, reach the global optima. Similarly, Bandyopadhyay et al. [34] determined the vital features of COVID-19 Computed Tomography (CT) scans utilizing Harris Hawks optimization with Simulated Annealing, a two-stage pipeline. However, their proposed method could be more computationally expensive. Recently, a transfer function has been proposed that works as a helper function of particle swarm optimization to determine the shape of a population [35]. Additionally, an improvement was proposed using Harris hawks optimization in [36] to make a hierarchy of features to convert a problem into an NP-hard problem and solve it iteratively. Finally, we have observed that all the metaheuristic approaches use nature-inspired optimization algorithms to improve their feature mining strategies for effective feature selection. However, they will always be task-dependent, and no generic methods exist. Depending on the task, there is a need to select optimization techniques that increase the algorithm search space. Moreover, all the problems are either NP-hard or NP, so they have exponential time complexity in the worst cases.

### 1.2. Objectives and Proposed Outcomes

All the recent dimension reduction techniques discussed in Section 1.1 are concerned with the physical interpretation of datasets. However, it is not feasible for them to eliminate genes based on only the physical interpretation of a dataset as they are only concerned with the data’s structure and their numeric representation. Therefore, both the biological and physical interpretation of these data are considered to eliminate unwanted genes. However, some recently advanced methodologies [37,38] have focused on analyzing gene expression data based on several factors (DNA methylation, DNA transcription, the transfection of gene vectors, cellular differentiation, and cell–cell interaction) attached to biological interpretation. Still, researchers have yet to try to use these studies to select the most informative genes. Ontological gene information and the protein–protein interaction network have been used for gene classification and protein synthesis in DNA methylation work, respectively.

**Figure 1:**
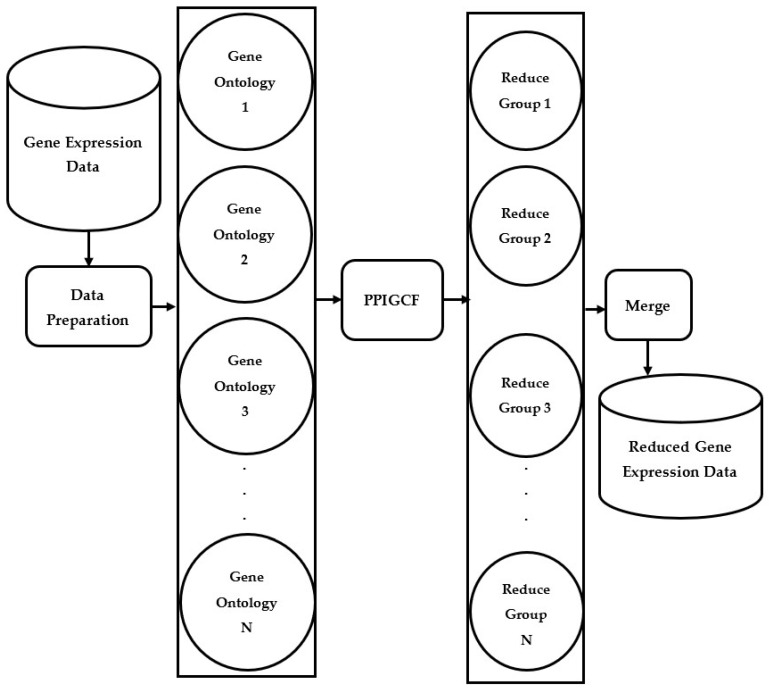
Brief flowchart of the proposed framework PPIGCF.

To overcome the above drawbacks, we propose protein–protein interaction-based gene correlation filtration (PPIGCF), which aims to identify the most informative genes concerned with a specific genomic disease while considering all the constraints. Here, combined information from physical and biological interpretation studies is utilized in a single genomic database for predicting the outputs with the help of molecular interactions and cellular process, which consolidates the novelty of the PPIGCF method. Figure 1 describes the main aim of this work. Here, the proposed method takes gene expression data as the input and classifies it into several gene ontological groups with the help of a gene ontological database. Then, the PPIGCF method examines the enriched signals of the genes in every group based on protein–protein interaction (PPI), the gene correlation coefficient (GCC), and information content (IC). Finally, a single gene expression dataset with higher classification accuracy is reproduced from all the reduced gene ontological groups.

### 1.3. Organization of Paper

This paper is structured into four sections. Section 2 gives a detailed description of the methodology and algorithm used to select informative genes. The result analysis and the comparative study are described in Section 3. Finally, the conclusion of the proposed work, with a brief discussion and an outline of the future scope of this topic, is described in Section 4.

## 2. Gene Selection Methodology

In this section, we describe PPIGCF, a dimension reduction method used to select the optimal number of informative genes from microarray data with maximum classification accuracy. PPIGCF has three defined layers. After passing all the layers, the experimental microarray data are ready to be analyzed for further experiments using the most significant genes, and for classification of the samples with reasonable accuracy by some well-known classifiers. Therefore, PPIGCF increases computational power regarding the experimental outcome, and computational time.

### 2.1. Dataset Preparation

This section describes the preparation of the dataset from the raw data to enable selection of the most informative genes from the microarray data [39]. The experimental dataset is an (n×m) matrix, where rows consist of the names of the genes $\left(g_{1},g_{2},\ldots ,g_{n}\right)$ and columns consist of the names of the samples ${(s}_{1},s_{2},\ldots ,s_{m})$. The names of the genes are extracted in this step to create a character vector consisting of all gene names. The gene ontology data are essential, corresponding to every gene expression before proceeding to the next step of the defined algorithm. Here, a map is drawn between the gene expression dataset and GO annotations for humans, and the corresponding GO annotation data are acquired. The mapping is performed using Equation (1).

(1) $$f\left(x,y\right)=y\cup x:\forall x\in G,\forall y\in GO,x\to y,x\cap y\neq \emptyset$$

where $f\left(x,y\right)$ is the mapping function, x represents the genes belonging to gene expression data (G), and y represents the GO annotation terms, including the biological processes (BPs) related to their cellular component (CC) domains in the GO database [40]. It performs one-to-one mapping between the experimental dataset and human GO annotations. This simple mathematical function works remarkably well, and as the method is constrained with time complexity, the proposed equations show linear time complexity. In contrast, the other mapping function is generally performed with quadratic time complexity.

Then, the process is ready to move to the next step, whereby the genes are grouped using the obtained GO dataset. In the obtained data, the vector contains the names of the genes, and the genes are classified based on the BPs corresponding to their CCs. Here, BPs and CCs are considered in the proposed PPIGCF method, where the CC information is used to classify the genes into several ontological classes, and BP information is used to track the mutation of genes. PPIGCF identifies each biological process corresponding to its cellular components and uses it as a classification parameter. Since we are considering biological processes related to cellular components, it is a viable gene ontological method to perform classification with high accuracy based on this parameter. After this, character vectors are obtained that are related to every CC. Here, the main advantage is that a gene cannot be part of more than one vector. So, the chance of gene duplicity is removed for the rest of the process. The following algorithm (Algorithm 1) defines the procedure for the classification of genes based on the BPs corresponding to their CCs.

**Algorithm 1**. Gene Ontology (GO) Classification***Input****: GO annotations with gene symbols* $\left(g_{1},g_{2},\ldots ,g_{n}\right)$.***Output***: *Characteristic vector of gene symbols corresponding to every cellular process*.*Begin*:
1.*Initialize GO index library*.2.*Find the GO index corresponding to the genes*.
a.*Gene:= Extract the GO terms. // GO groups*b.*Find their corresponding GO information content using Equation (1)*.3.
*For each Gene, do the following:*
a.
*Beach:= gene list corresponding to every GOIC (GO Information Content).*
b.
*Set GO_ID as a cellular component.*
i.
*For each cc(j) ∈ Cellular Component do: // g(i): genes ∈ GO Group*
1.
*If (g(i) ∩ Beach)) then: //If the gene present in the GOIC*
*cc(j)* ∪ *= g(i); //then merge them as a participating*2.
*End if. //genes of that cellular component*

ii.*End for*.
c.*End for*.
4.*End for*.5.
*Set Ontology:= GO.*
6.*Set IC = GOIC.// Setting the gene ontology reference*.7.
*For each Ontology(i) do:*
a.
*For each IC (j) do:*
i.$Sim_{ij}:=Ontology\left(i\right)*IC\left(j\right);$*//Element-wise multiplication*.
b.*End for*.
8.*End for*.9.*Return* $Sim_{ij}$;
*End*.

Now, the experimental dataset is divided into nine ontological gene groups corresponding to six biological processes (ABP (*Androgen-Binding Protein*), AMF (*Agro Marker Finder*), ACC (*Amino Cyclopropane Carboxylate*), MBP (*Myelin Basic Protein*), MMF (*Mycophenolate Mofetil*), and MCC (*Maternal Cell Contamination*)). This GO is a candidate colorectal tumor suppressor gene that is thought to negatively regulate cell cycle progression. The orthologous gene in mice expresses a phosphoprotein associated with the plasma membrane and membrane organelles, and overexpression of the mouse protein inhibits entry into the S phase. Multiple transcript variants encoding different isoforms have been found for this gene.

Additionally, a similarity matrix ($Sim_{ij}$) is obtained, which will help find the PPIs in every gene ontological group in the next step. However, several GO classes in the GO library concerning several BPs correspond to their CCs. According to Zhang et al. [41], there are five billion biological processes related to the human cancer genome; however, the gene expression dataset used for experiments is mainly affected by the six BPs mentioned above corresponding to their nine CCs. Algorithm 1 is a fully automated process with no human intervention or knowledge employed for this classification. Additionally, if the experimental dataset changes, it can automatically detect the affected BPs related to their CCs (https://github.com/ayanbabusona/jNMF/blob/master/genesim.R; accessed on 26 June 2019).

### 2.2. Protein–Protein Interactions

The genes are classified according to their active participation in cellular processes at this stage. However, molecular-level information is also needed to know the genes’ involvement in the disease. This method determines the protein–protein interactions to describe molecular-level information. We propose PPIGCF as a novel algorithm to obtain this PPI of every cellular component. In this method, no prior Human Protein Interaction Database [42] is needed to obtain the PPI data corresponding to every GO class, because the knowledge of graph theory is utilized in Algorithm 2, which shows the novelty of the proposed method. It is observed that the pattern-matching algorithm using the PPI database is quite expensive in terms of resources. The proposed method obtains all possible combinations of BPs corresponding to the CCs in which the genes have participated in finding an alternative pathway. Then, based on those interactions, a weighted adjacency matrix is obtained. Let two genes, $g_{a}$ and $g_{b}$, participate in a BP corresponding to a CC. Compute their correlation; if they have a high correlation, they are likely to participate in PPI interactions. So, the adjacency matrix obtains a value of 1. This is a proposed probabilistic approach to reducing the complexity of obtaining a PPI network. Essentially, PPI data represent an adjacency matrix where a 1/0 entry represents the presence/absence of interactions. If a gene does not participate in a protein–protein interaction, then this gene can be eliminated from the informative gene list. Algorithm 2 describes the process of finding the PPI interaction of every cellular component.

**Algorithm 2.** PPI interactions***Input:*** *Data containing gene names related to their cellular process and expression value*.***Output:*** *PPI networks*.*Begin*:
/* To find weight matrix w.r.t.

$Sim_{ij}$

*/

/* n = gene number, m = sample number */

/*

$d_{ij}$

= input data matrix */

1.
*for each i:=1 to n do:*
a.
*for each j:=1 to m do:*
i.$W_{ij}≔d_{ij}\times Sim_{ij}$;
b.*End for*.
2.*End for*.
/* To find the interactions of genes from $W_{ij}$ and store in the corresponding list*/
3.
*for each i:=1 to n do:*
a.
*for each j:=i + 1 to m do:*
i.$AdjList_{i}:=W_{ij-1}$;ii.*If (*$W_{ij-1}\to W_{ij}$*) then: //If the edge present between* $W_{ij-1}$ *and* $W_{ij}$1.$AdjList_{i}:=AdjList_{i}\cup W_{ij}$;iii.*End if*.
b.*End for*.
4.*End for*.5.*Remove the occurrence of duplicate genes from AdjList*.6.*Draw a graph (G) from the AdjList that shows the participant gene’s interaction*.7.
*If (* $G(v_{i},v_{i+1}$ *) == 1) then:*
a.*Collect the respective genes and add them to*$RD_{1}$.
8.
*Else*
a.*Eliminate both genes*.
9.*End if*.

*End.*


Here, $AdjList_{i}$ is an adjacency list that keeps track of protein–protein interactions initialized with zero. We add the corresponding gene expression value to the list whenever a gene interaction occurs. The data frame $RD_{1i,i=1,2,\ldots ,9}$ contains the genes participating in the PPI interactions. Algorithm 2 is run for all nine groups and obtains the $RD_{1i,i=1,2,\ldots ,9}$ corresponding to each group. This $RD_{1i,i=1,2,\ldots ,9}$ is the input data frame for the next step.

### 2.3. Gene Correlation Filter

After finding the PPI of every cellular component, this method eliminates insignificant genes whose interactions are absent in the network. Here, genes that are strongly correlated with each other are obtained from protein–protein interaction networks. However, the genes that survive after the PPI-based elimination are not the only essential genes that may cause the disease. There are still some noisy genes that may affect performance at a later stage. During the mutation process, there is a possibility that part of the genome may be affected during transformation and transcription when the mutation affects critical gene regulation or essential function, and these types of genes are rejected. Otherwise, the computational cost of analyzing noisy genomes is increased. Therefore, gene correlation filtration aims to select weakly correlated genomes and eliminate them from the network. The methodology is elongated using the Normalized Square Correlation Coefficient (NSCC) [43] to obtain strongly correlated genomes. Firstly, we identify an n × n pairwise Pearson’s Correlation Coefficient (PCC) matrix using Equation (2).

(2) $${PCC}_{x,y}=\frac{\sum_{i=1}^{n}{(x}_{i}-\mu_{x})\times (y_{i}-\mu_{y})}{\left(n\times \sigma_{x}\times \sigma_{y}\right)}$$

Here, μ and σ are the mean and standard deviation, respectively. Suppose that any gene entry in the matrix gives a negative PCC value. In this case, it would be removed, as negative correlation values demonstrate inverse relationships [44], affecting the classification performance of our algorithm. Additionally, the negative correlation coefficient is directly proportional to the degree of correlation between respective genes, which shows the strongly inverse topological properties of the genes that should be eliminated. Thus, those genes are removed from the data as they are considered insignificant genes. Thus, based on the correlation coefficient values, some genes can be eliminated, and then, the gene rank is computed using Equation (3) for the rest of the genes.

(3) $$R_{ij}^{2}=\frac{\sum_{i=1}^{n}r_{ij}^{2}}{\sum_{k=1}^{n}r_{ik}^{2}}$$

where *R* and *r* are the NSCC matrix and the PCC matrix, respectively. If two genes have positive connectivity in the PCC and their NSCC tends to zero, then the genes (such as $g_{i}$ and $g_{j}$) are strongly correlated with each other.

In addition, PPIGCF performs biological interpretation of this algorithm to obtain the gene correlation filter. Moreover, it checks for functional and semantic similarity, as described in [45]. Then, the functional similarity of the likelihood score is calculated using Equation (4).

(4) $$LLscore_{i}\left(g_{i},g_{j}\right)=\frac{R_{ij}-R_{ij_{min}}}{R_{ij_{max}}-R_{ij_{min}}}$$

The $LLscore_{i}$ represents the functional similarity of the ith gene, $R_{ij}$ is the rank of the ith gene compared to the jth gene in the NSCC matrix, and $R_{ij_{min}}$ and $R_{ij_{max}}$ are the minimum and maximum gene ranks in the NSCC matrix, respectively. Moreover, Equation (5) is used to obtain the semantic similarity.

(5) $$Semsim\left(g_{i},g_{j}\right)=\frac{{(g}_{i}\times g_{j})}{\mu_{g}^{2}}$$

where $\mu_{g}^{2}$ represents the mean value of the gene expressions. Algorithm 3 describes the gene correlation filter method.
**Algorithm 3.** Gene correlation filter (GCF)***Input:*** $RD_{1}$***Output:*** *Strongly correlated genes in the PPI network*.*Begin*: *Compute Pearson’s correlation matrix (*$r_{ij}$*) using Equation (2*). *for each i = 1 to n do:*  *for each j = 1 to n d*o:   *if*
$r_{ij}$ > *0 then:*    *Compute*
$R_{ij}^{2}$
*using Equation (3)*.    *if*
$R_{ij}\to 0$
*then:*     *if*
$LLscore_{i}\left(g_{i},g_{j}\right)\to 1\&\&Semsim\left(g_{i},g_{j}\right)\to 1$
*then:*      $RD_{2}\cup R_{ij}$;     *End if*.    *End if*.   *else*:    *Eliminate (*$g_{i},g_{j}$*);*   *End if*.  *End for*. *End for*. *return* $RD_{2}$;*End*.

$RD_{2i,i=1,2,..,9}$ is the reduced dataset after performing Algorithm 3. The correlated genes are eliminated after this step, which affects protein–protein interactions. This step is repeated for the $RD_{1i,i=1,2,..,9}$ of all nine groups created using Algorithm 2.

### 2.4. Significance of Information Content

In this step, the most significant genes in a cancer dataset are obtained. After running the gene correlation filter algorithm (Algorithm 3), the method obtains a strongly correlated gene set at the molecular level, i.e., the genes form a strong PPI network. The primary objective of the proposed method is to reduce the number of insignificant genes in the experimental data to achieve maximum classification accuracy. For this reason, the significant information content (IC) values are computed, and a GO analysis table is required, which describes the six gene ontology analysis (GOA) [46] methods used to obtain the IC threshold value [47]. Then, the IC value is computed for every identified gene in the previous step. This value is compared with the mean IC threshold value (δ) of the six techniques. If the computed IC value is greater than δ, then the respective gene is taken as the most significant gene. This procedure is performed repeatedly for all identified strongly correlated genes in the PPI network, which form the reduced dataset. Here, the Codon Efficiency Term (CET) (defined in Equation (6)) of each gene is required to find the IC.

(6) $$CET\left(g\right)=Specificity(g)\times Coverage(g)$$

where

(7) $$Specificity\left(g\right)=1-\log\left(g\right)+\frac{1}{\sum_{i=1}^{n}\log\left(g\right)}$$

and

(8) $$Coverage\left(g\right)=1-\frac{\sum_{i=1}^{n}g^{2}}{n\left(n^{2}-1\right)}$$

where $Specificity\left(g\right)$ denotes the depth of the gene g in its corresponding GO hierarchy, and the maximum depth of the gene g is taken as its depth, as depicted in Equation (7). Similarly, $Coverage\left(g\right)$ measures the dependency fraction of the gene g to its descendants in GO. The terms at lower levels are more specific to a larger IC, while the terms with a smaller IC have more descendants and are more general.

Algorithm 4 computes the IC value for each gene of all $RD{}_{2}$, and this computation is needed for the functional definition of associative terms (Acute Similarity (AS) and IC threshold value).

Let B be the bipartite component of $RD_{2}$, $B^{*}$ be the closure of set B, f(B) denote the highest frequency occurrence of a gene g in $RD_{2}$, and $X_{B}\left(g\right)$ represent the relative frequencies of the involved bi-partitions; then, the terms $X_{B}\left(g\right)$ and $X_{B^{*}}(g)$ are defined in Equations (9) and (10).

(9) $$X_{B}\left(g\right)=\frac{f\left(B\left(g\right)\right)}{f\left(B\left(g\right)\right)+f(B^{*}(g))}$$

and

(10) $$X_{B^{*}}\left(g\right)=\frac{f\left(B^{*}\left(g\right)\right)}{f\left(B\left(g\right)\right)+f\left(B^{*}\left(g\right)\right)}$$

Here, $X_{B}\left(g\right)$ and $X_{B^{*}}\left(g\right)$ are complementary to each other, and *f()* is the function used to compute the relative frequencies of the gene g among all the bipartite graphs obtained from the PPI network in which the gene g participates. Using this information, the IC value of the gene g is computed using Equation (11).

(11) $$IC\left(g\right)=\frac{1}{\lambda }(X_{B}\left(g\right)\times \log_{2}X_{B}\left(g\right)+X_{B^{*}}\left(g\right)\times \log_{2}X_{B^{*}}\left(g\right))$$

where λ is the normalization hyperparameter obtained through the hyperparameter grid search. The conflicting set C(g) (defined in Equation (12)) of a gene is computed by partially differentiating the cross-product of the two complementary matrices (i.e., $X_{B}\left(g\right)$ and $X_{B^{*}}\left(g\right))$. Basically, C(g) is a type of Jacobian matrix.

(12) $$C\left(g\right)=\frac{\partial }{\partial g}(X_{B}\left(g\right)\times X_{B^{*}}\left(g\right))$$

C(g) is required to compute the Acute Similarity (AS) of the respective gene g in $RD_{2}$ [48]. The AS of a gene g is defined in Equation (13). The AS is for the associate gene set of the matrix and helps to determine the topological property (one of the most important biomarkers of gene selection) of the corresponding gene *g.*

(13) $$AS\left(g\right)=1+\sum_{g\in RD_{2}}\log_{2}\left(X_{C\left(g\right)}\times \log_{2}X_{C\left(g\right)}\right)$$

In this step, the GO analysis (GOA) [47] with the metrics ECC, RES, SEQ, and Pfam is required to obtain the IC threshold (defined in Equation (14)). Here, all six GOAs are considered as every GOA sets a different threshold based on its experimental parameters. As all the genes participate only in these six GOAs, this method considers their weighted means to determine the IC threshold.

(14) $$IC_{threshold}=\frac{1}{x}\times \sum_{i=1}^{x}GOA_{i}$$

where x is the number of metrics in the GOA. The IC term of each gene is computed using Algorithm 4.
**Algorithm 4.** Find IC values***Input:*** *Strongly correlated genes in the PPI network *($RD_{2}$).***Output:*** *The genes with IC value*s.*Begin*:  *Find the CET of g using Equation (6)*.  *for each*
$g_{i}\in CET\left(g_{i}\right)$
*do:*    *for each*
$g_{j}\in CET\left(g_{j}\right)$
*do*:      $IC\left(g_{i}\to g_{j}\right)=IC\left(g_{i}\right)-IC\left(g_{j}\right)$;    *End for*.    $IC\left(AS\left(g_{i}\right)\to g_{i}\right)=IC\left(g_{i}\right)-IC\left(AS\left(g_{i}\right)\right)$;  *End fo*r.  *return*
$IC\left(g_{i}\right)$;*End.*

After obtaining the IC value of each gene $\left(IC\left(g_{i}\right)\right)$, the most significant genes are obtained and compared to the IC threshold ($IC_{threshold}$). If the IC value of any gene is greater than the IC threshold, this gene is selected as the most significant gene. Otherwise, the respective gene is removed from the reduced dataset.

This method obtains the optimal number of genes from every gene ontological group (GO group). Then, the nine GO groups are merged based on the identified genes to form the final reduced gene subset containing all of the most significant genes identified in the workflow.

### 2.5. Overall Proposed PPIGCF

**Figure 2:**
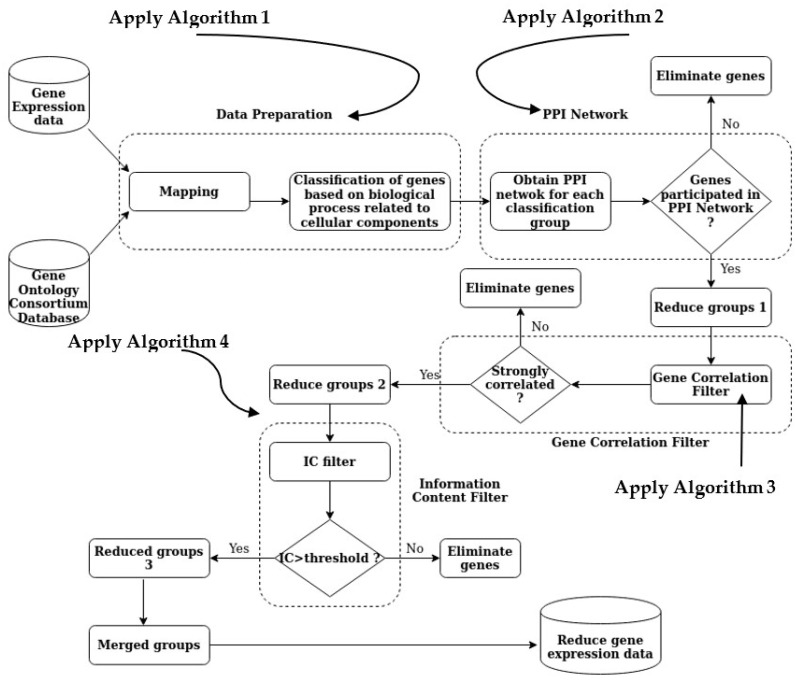
The overall methodology of PPIGCF.

The PPIGCF method obtains a summarized version of the dataset, which contains the most significant genes, after completing all the steps of the proposed method (Figure 2). The following section provides a detailed description of the performance evaluation of the PPIGCF method to check whether the reduced data are replicas of the original experimental data based on high classification accuracy.

## 3. Results

Our experiments were conducted on several publicly available benchmark microarray data with a high volume of insignificant genes, and linearly inseparable samples were taken from the Kent Ridge Biomedical Dataset Repository [49]. Descriptions of all the used gene expression datasets are listed in Table 1.

### 3.1. Experimental Setup

The proposed methodology was implemented using RStudio IDE with dedicated R programming. It can run on desktop (Linux, Windows, and Mac) or in a browser linked to RStudio Server Pro/RStudio Server (Ubuntu, Red Hat, and SUSE Linux). The proposed methodology and all the comparative approaches were analyzed in an Ubuntu-based OS with 4 GB RAM and an Intel i3 processor. All the performance analyses were performed using edge with the Bioconductor package. The code is available at https://github.com/ayanban011/HandsonML/tree/main/bioinformatics; accessed on 18 June 2021.

### 3.2. Performance Evaluation of PPIGCF

PPIGCF is a stepwise progression process used to reduce the dimension of microarray data. All the stepwise experiments and evaluations were performed as described in the following subsections.

#### 3.2.1. Grouping of Genes Based on Ontological Similarity

Firstly, the gene names were extracted from biological data sources. These data were classified based on the BPs related to their CCs. The GO behavior of the genes played an essential role in their classification. Here, PPIGCF obtained nine GO classes with their GO IDs, cellular components, and the number of genes in every category for each cancer datapoint. Only nine groups (with GO IDs: GO:0003674, GO:0005764, GO:0005783, GO:0005794, GO:0005886, GO:0008150, GO:0016021, GO:0005737, and GO:0015630) were considered because these are the most-affected classes when a normal cell transforms into a tumor cell. The details of these nine groups are listed in Supplementary Materials Table S1.

From Supplementary Materials Table S1, it can be concluded that prostate cancer exhibited a symmetric probability distribution, whereas leukemia, colon cancer, DLBCL, and lung cancer exhibited an asymmetric probability distribution over the nine groups.

#### 3.2.2. Elimination of Genes through PPI

In this step, the protein–protein interactions of each gene ontological group are obtained, and the genes that did not participate in the PPI interactions are identified. These genes are referred to as isolated interaction genes (IIG) and were treated as insignificant genes in further experiments. Therefore, these genes were eliminated from the data. The result of this step for all data are listed in Supplementary Materials Table S2.

**Figure 3:**
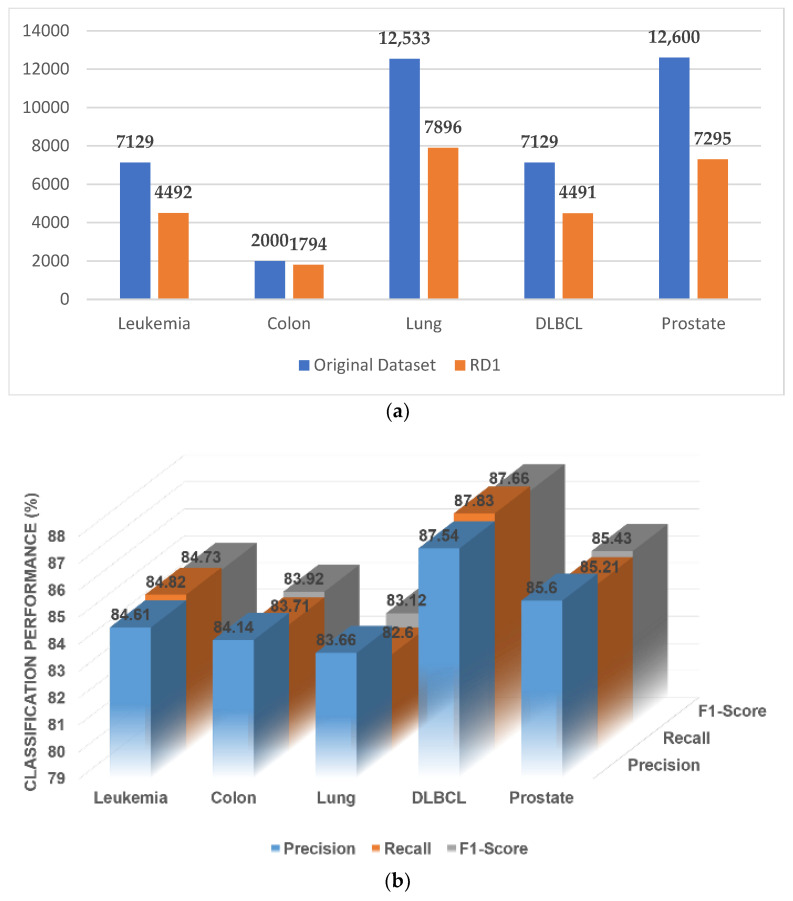
(**a**) A comparative study with the number of genes before and after the elimination. (**b**) Classification performance of the original data.

Figure 3a gives a brief idea of the number of genes reduced after this step of elimination, and the classification performance (note: only the classification performance of the SVM is reported here and in the following figure) on the reduced set of genes is given in Figure 3b. The protein–protein interactions of the GO:0003674 group of all five datasets (leukemia, colon cancer, DLBCL, lung cancer, and prostate cancer) are shown in Supplementary Materials Figure S1a–e, respectively. The remaining eight ontological groups also formed these networks based on Algorithm 2.

In Supplementary Materials Figure S1a–e, some genes remain isolated in protein–protein interactions. These genes can be treated as dead genes, as they lose their transcriptomic property and would not be further mutated to convert a normal cell to a tumor cell. These genes should be eliminated to reduce the duration of the genomic therapy treatment.

Here, the colors represent the similarity of the genes. Genes of the same color hold similar GO properties. Additionally, the colors of the edges represent the strength of the connectivity. If the edges are green, this suggests a strong correlation between the two genes. In contrast, if two genes are connected by red edges, they have a very weak correlation and may be isolated in the later reduction stages.

From Supplementary Materials Figure S1f, the overall classification performance increases for all the experimental datasets, as the expression level, related function, and topological properties of these remaining genes are very similar after each step of elimination. This was the ultimate motivation for conducting this experiment, and this result shows the importance of gene selection from microarray data while considering biological interpretation.

#### 3.2.3. Elimination of Genes through Correlation Filter

PCC was calculated for the remaining genes. If any gene entry gave a negative PCC value, this gene was removed from the data as an insignificant gene because these genes provide less correlation. After this, the NSCC was calculated for every gene, and if the NSCC value tended toward 1, then the respective gene was insignificant and removed for the rest of the step. The detailed results of this step are listed in Supplementary Materials Table S3, where a significant number of the genes are removed from the previous step.

**Figure 4:**
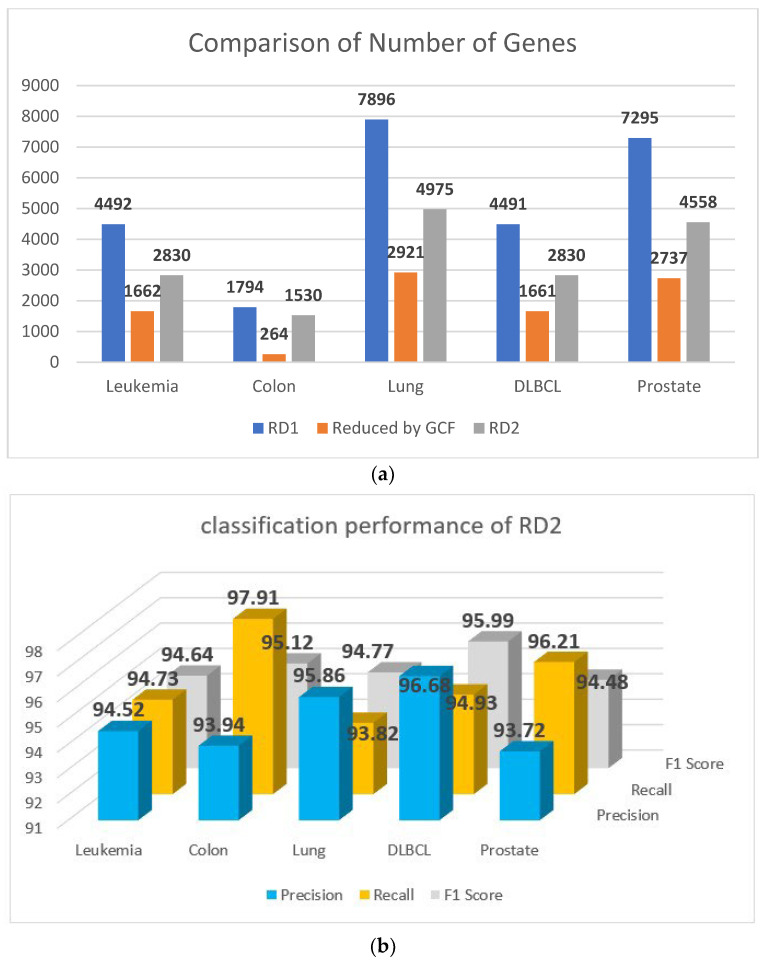
(**a**) Comparison of the number of genes before and after elimination. (**b**) Classification performance of RD2.

Additionally, Figure 4a shows the volume of the genes before and after applying this step of elimination, and the classification performance of this step is reported in Figure 4b.

#### 3.2.4. Elimination of Genes through Information Content

In this step of eliminating PPIGCF, the IC values were computed for all genes of the reduced data. Here, six gene ontology analysis (GOA) methodologies (namely, ABP, AMF, ACC, MBP, MMF, and MCC) were performed based on four Correlational Estimations of Semantic Similarity Measurement (CESSM) [50] metrics (namely, ECC, RES, Seq, and Pfam). Moreover, the IC values were obtained for every metric (shown in Table 2).

**Figure 5:**
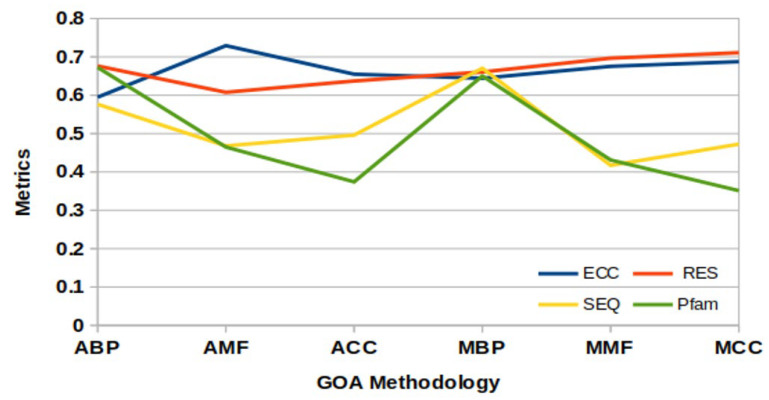
IC threshold values from Gene Ontological Analysis.

Figure 5 visually compares the IC values computed by the six GOA methods under four different parameters.

After this, the $IC_{-threshold}(\delta )$ was computed using Equation (13) for PPIGCF. Here, δ=0.6637. The δ=0.6637 point is shown in Figure 5, where all the curves are supposed to intercept. Genes whose computed IC values were lower than δ were eliminated, and the remaining genes were selected as the most informative genes for specific data. Therefore, the numbers of the most informative genes for each experimental dataset after passing all the steps of the PPIGCF method are listed in Supplementary Materials Table S4.

**Figure 6:**
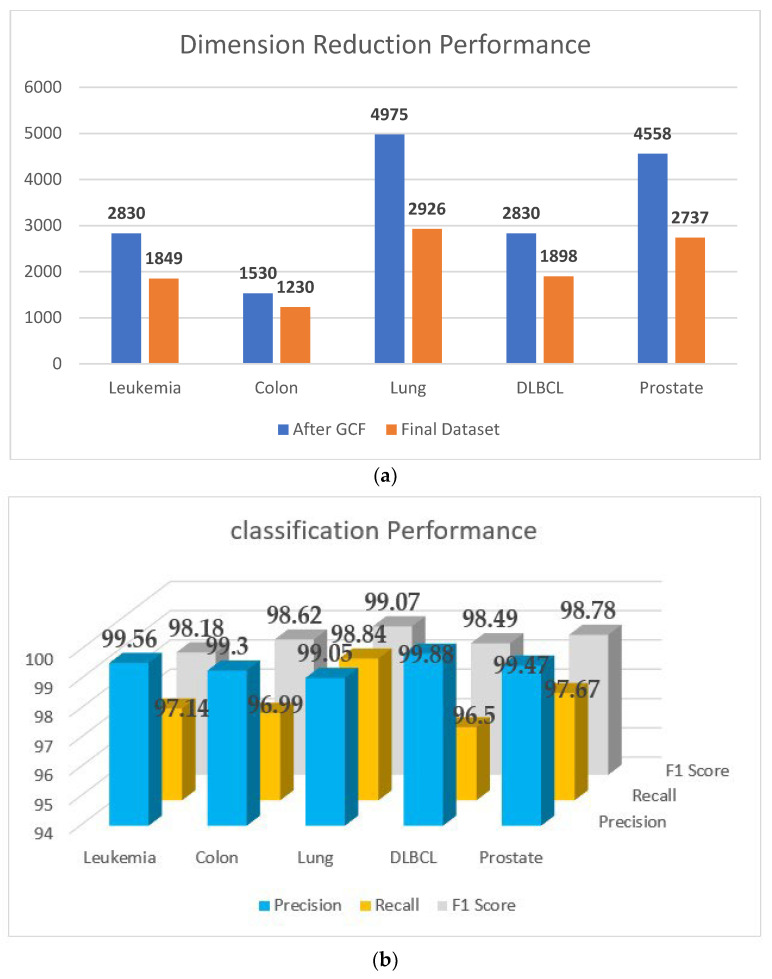
Final outcome of PPIGCF: (**a**) dimension reduction performance; (**b**) classification performance.

A visual representation of the outcome of the proposed PPIGCF for all experimental datasets is shown in Figure 6, along with the classification performance.

#### 3.2.5. Classification Performance

The most informative genes were collected after completing all the filtration steps of PPIGCF and forming a replica (reduced data) of the original dataset. Here, four commonly used classifiers (namely, Nearest Neighbor (KNN) [51], Random Forest (RF) [52], Support Vector Machine (SVM) [53], and Naïve Bayes Classifier [54]) were utilized to measure the classification performance of the reduced dataset and original data, listed in Table 3. The hyperparameter descriptions of each of the classifiers are as follows:

1. **K-Nearest Neighbor (KNN).** K: the nearest neighbors (here, k = 20). Distance metric: Mahalanabis distance metric is used to calculate distance.
2. **Random Forest (RF).** n_estimators: the number of decision trees to build in the forest (here, 20). max_features: the maximum number of features to consider when splitting a node in a decision tree (here, 100). max_depth: the maximum depth of a decision tree (here, 15). criterion: the function used to measure the quality of a split (Gini impurity).
3. **Support Vector Machine (SVM).** Kernel: the function used to transform the data into a higher-dimensional space (here, radial basis function (RBF)). C: the penalty parameter for misclassifications (here, 0.01). Gamma: a kernel coefficient for the RBF kernel (here, 0.1).
4. **Naïve Bayes Classifier (NBC)**. Smoothing parameter: the parameter used to smooth the probabilities (here, Lidstone smoothing). Distribution: the probability distribution used to model the data (here, multinomial distribution).

All the hyperparameters were fixed through the hyperparameter grid search. Here, the k-fold cross-validation method was used to calculate the accuracy, and the average accuracy values are listed in Table 3 with suitable k values (k = 10) for different classifiers.

We used this technique and bootstrapping to estimate the model’s performance on experimental data. These techniques involved randomly splitting the data into training and validation sets and repeating this process 50 times to obtain a more robust estimate of the model’s performance.

Table 3 shows that the FRD formed by PPIGCF is a replica of the original data based on high classification accuracy. Moreover, it is proven that if any microarray dataset is passed through all steps of the PPIGCF method, the reduced data are formed with the most informative genes related to a specific disease.

**Figure 7:**
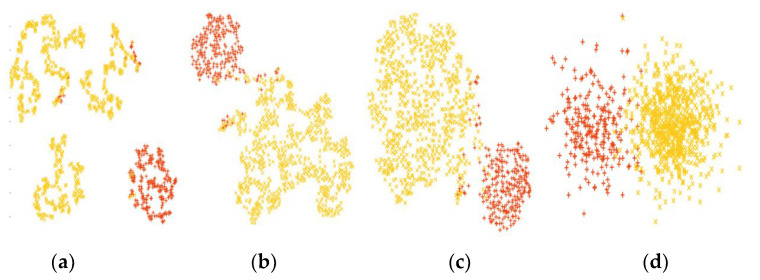
t-SNE distribution (red and yellow colors represent the AML and ALL samples, respectively) of the leukemia dataset. (**a**) Original dataset, (**b**) RD1, (**c**) RD2, and (**d**) final reduced dataset.

In order to establish the fact that PPIGCF performed a stepwise gene elimination process that only selected informative genes as a final dataset, and that the classification performance also increased in each step, a data distribution map of the leukemia dataset is shown in Figure 7.

It can be observed from Figure 7 that the data distribution gradually increases for both classes from the original to the final reduced dataset. In Figure 7a, the genes are distributed irregularly, whereas in Figure 7d, the genes are clustered in two groups. This figure also provides evidence, as shown in Table 3, that the classification performance of each classifier is similar for the original dataset; however, a regular pattern (with SVM achieving the highest classification performance) is observed after PPIGCF for each of the classifiers, which establishes the novelty of the proposed method.

#### 3.2.6. Identification of Biological Significance

The most informative gene subset (reduced data) was collected from the experimental dataset after applying the proposed PPIGCF. KEGG pathway analysis was performed on the reduced data to show the biological significance of the outcome of the PPIGCF method. This representation focuses on the network of gene products with functional RNAs. The obtained genes that were well connected in the FRD were put through KEGG pathway analysis using shinyGo.

Table 4 describes the KEGG pathways that were mapped to leukemia genes obtained from the FRD. The Significance column explains why these two pathways explain are biologically crucial to identifying leukemia from the microarray dataset. Arachidonic acid metabolism and pancreatic secretion were selected from the BRITE hierarchy. The BRITE hierarchy is a modern classification system for KEGG pathways, with objects identified using a KEGG identifier.

Table 5 shows the significance of KEGG pathways found via KEGG mapping using shinyGO on colon cancer genes obtained from the FRD using PPIGCF. Both pathways were derived from the BRITE hierarchy.

Table 6 describes the role of KEGG pathways that are mapped to lung cancer genes obtained from the FRD. In the complement and coagulation cascade pathway, we mapped two significant genes that are most affected in the pathway. The three pathways were selected from the KEGG BRITE hierarchy. Their fold change score gives a significant idea of their participation in the development of lung cancer.

Table 7 describes the significance of KEGG pathways that are mapped to DLBCL genes obtained from the FRD. The four pathways were obtained from the BRITE hierarchy. Among the pathways, aldosterone-regulated sodium reabsorption is an excretory system, while the P53-Signaling pathway is essential to blocking tumor progression and the growth of cancer cells.

Table 8 explains the role of two pathways, namely biosynthesis cofactor and tryptophan metabolism, and their roles in cancer progression. Both pathways belong to the KEGG BRITE hierarchy. Tryptophan metabolism has a higher fold change score, which indicates that it may be more dominating than the biosynthesis of cofactors in the case of prostate cancer.

### 3.3. Comparative Study and Performance Analysis

It is concluded that PPIGCF does not change the physical (expression values) and biological interpretation of data, such as ontological behavior, PPI network connection, DNA methylation, DNA transcription, and data translation; only the dataset size is reduced. This meets the desired goal of the proposed method. Nevertheless, several dimension reduction methods have been proposed in the last five years. Our proposed method used several parameters to compare the performance of PPIGCF with recent papers [28,29,30].

#### 3.3.1. Comparison based on the Number of Genes

Table 9 shows the performance analysis comparing the number of genes and classification performance (CP), based on the accuracy (%), between papers [28,29,30]. These methods were chosen for the comparative study as these are the most recent papers published on the dimension reduction of microarray data. They were used to form a rationale and show gene ontology’s importance while considering the genes’ physical properties, in order to eliminate them.

In Table 9, it is shown that PPIGCF selects a smaller number of genes and has higher classification accuracy compared to the methods used in other papers [28,29,30]. It is shown that PPIGCF specifies fewer genes, reducing experimental time (marked in Table 5) and the cost of the diagnosis of diseases. Additionally, the methods proposed in [28,29,30] already outperformed other classic feature selection methods for microarray data mentioned in [69], so it can be concluded that PPIGCF is the most advanced feature selection method.

The application of PPIGCF in gene therapy and biomedicine is based on the idea that targeting a group of functionally related genes, rather than individual genes, may be more effective when treating complex diseases. By targeting correlated genes that work together, it may be possible to achieve a more significant therapeutic effect.

While it is true that the reduced set obtained by PPIGCF still contains more than 1000 genes, this is still a significant reduction from the thousands of genes in the human genome. Moreover, the genes within the correlation identified by PPIGCF are functionally related, which may facilitate the identification of potential therapeutic targets. In summary, the application of PPIGCF in gene therapy and biomedicine is based on the idea that targeting functionally related genes may be more effective than targeting individual genes. While the reduced set obtained by PPIGCF still contains more than 1000 genes, the functional relatedness of these genes may facilitate the identification of potential therapeutic targets.

#### 3.3.2. Comparison based on Experimental Time

Table 10 shows the performance analysis comparing the experimental time of PPIGCF with the methods used recent papers [28,29,30]. Table 5 shows that PPIGCF takes less experimental time than the other compared methods [28,29,30], with all computational times taken based on the experimental setup (described in Section 3.1). Furthermore, the PPIGCF algorithm takes $O\left(n^{2}\right)$ in worst-case time complexity. The methods mentioned in [28,29,30] are the only ones that run on a CPU; a GPU is required as a processing unit for the other techniques. So, these are the most straightforward and efficient methods selected for the comparative study.

Table 10 shows that the L1-regularized filter takes significantly less time (marked in bold text); however, the feature selected by the L1-regularized filter needs to reach state-of-the-art performance. However, PPIGCF takes comparatively less time (marked in bold text) than the other methods and performs better than the state-of-the-art performance level.

#### 3.3.3. Comparison based on Statistical Parameters

The performance analysis was conducted based on some statistical parameters, such as the True Positive Rate (TPR), False Positive Rate (FPR), Testing Accuracy (ACC), and f1-score (f1), and was computed using Equations (15)–(18).

(15) $$TPR=\frac{True\;Positive}{True\;Positve\;+\;False\;Negative}$$

(16) $$FPR=\frac{False\;Postive}{True\;Negative\;+\;False\;Positive}$$

(17) $$accuracy=\frac{True\;Positive\;+\;False\;Negative}{True\;Positive\;+\;True\;Negative\;+\;False\;Positve\;+\;False\;Negative}$$

(18) $$F1\;score=\frac{2*\left(Sensitivity\;*\;Specificity\right)}{Sensitivity+Specificity}$$

where True Positive is the class1 sample classified as class1, False Positive is the class1 sample classified as class2, True Negative is the class2 sample classified as class2, and False Negative is the class2 sample classified as class1. In the experimental microarray datasets, the samples are fully distinguished as class 1 and class 2 (referred to in Table 1). Therefore, the statistical metrics were efficiently computed using Equations (15)–(18).

**Figure 8:**
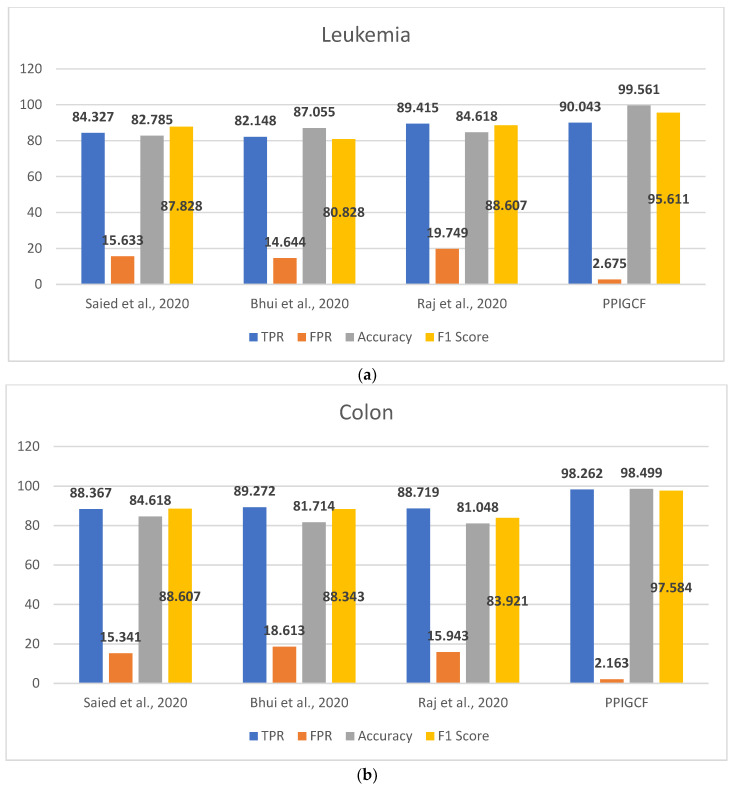

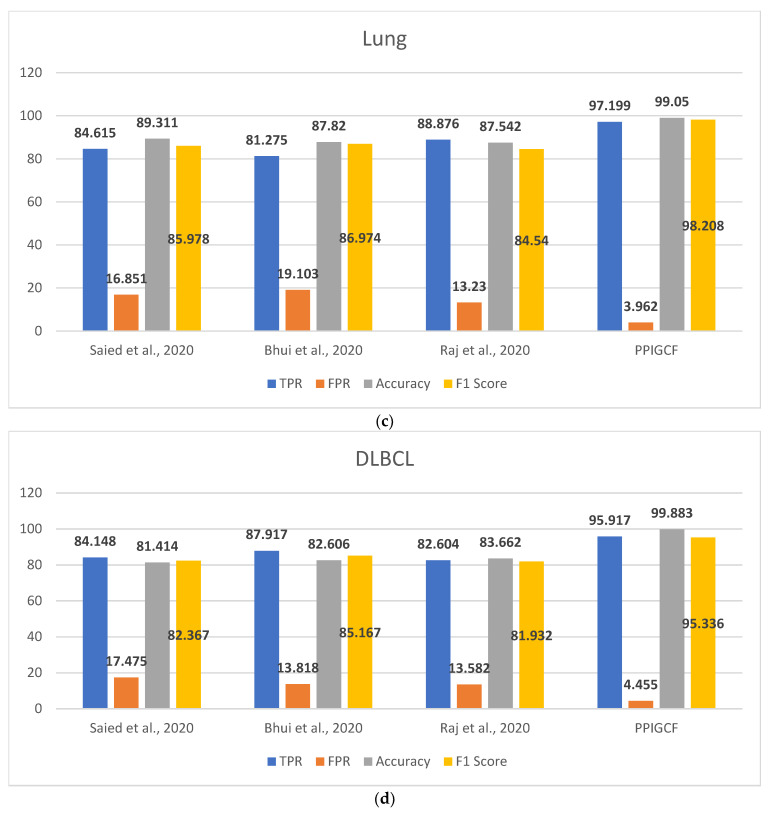

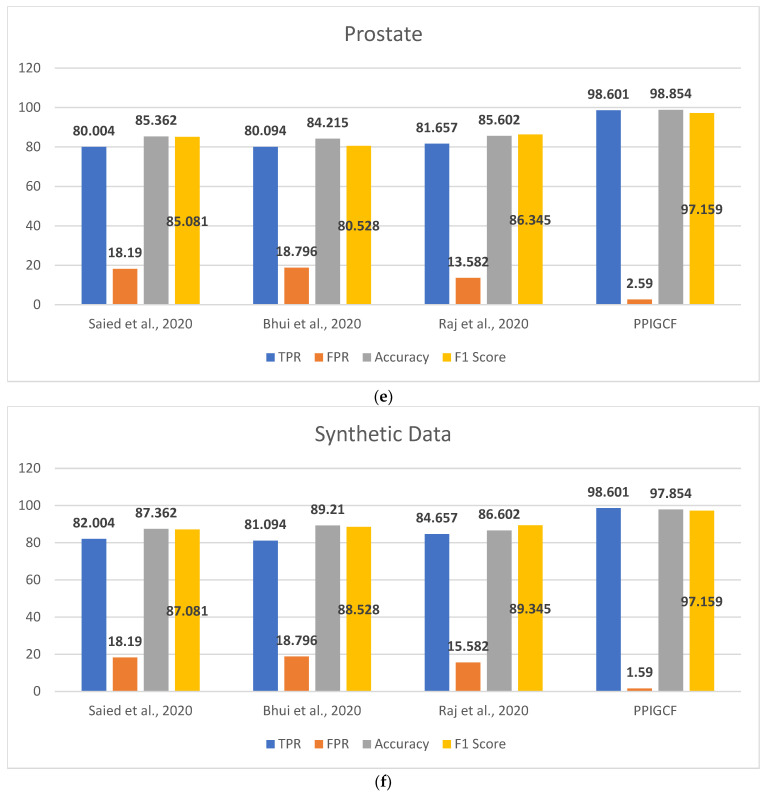
Comparative study of the experimental dataset: (**a**) leukemia, (**b**) colon cancer, (**c**) lung cancer, (**d**) DLBCL, (**e**) prostate cancer, and (**f**) synthetic datasets. [28,29,30].

Figure 8 shows the performances of PPIGCF and the compared methods [28,29,30] based on the used statistical parameters.

Figure 8 shows that PPIGCF performs better than the other compared methods based on the used statistical parameters. In Figure 8f, a synthetic dataset was prepared by combining the five experimental datasets. Each dataset has two classes (cancerous and non-cancerous) to check the effectiveness of the proposed PPIGCF on large datasets. PPIGCF also outperforms the state-of-the-art approaches. The reason for PPIGCF’s better performance is its feature selection of the biological interpretation of microarray data, which is an essential parameter, as it contains information related to the disease. According to the knowledge base, PPIGCF uses this biological interpretation to eliminate combining their physical properties for the first time. Because PPIGCF considers both biological as well as physical factors, it can be applied to any microarray dataset and gene sequencing data.

In general, PPIGCF selected the most informative genes from the microarray cancer data, which satisfies the predefined objectives, such as dimension reduction based on biological interpretation, high classification accuracy, and less computational time than the existing recent state-of-the-art approaches [28,29,30]; this indicates the effectiveness of the proposed PPIGCF.

Overall, PPIGCF is effective as it has generalization power. It ultimately depends on the biological interpretation of the genes present in the microarray data and solves the problem in quadratic time complexity (i.e., O(n^2^)). It helps eliminate the vast algorithm search space to select the best optimization strategy and reduce the exponential-to-quadratic complexity. Nevertheless, other state-of-the-art dimension reduction strategies depend on physical interpretation of the data (i.e., microarray value, probability density function, distribution, etc.), which may be effective for text or image feature mining, but not for genes. As shown above, PPIGCF can outperform state-of-the-art approaches by an adequate margin and sets a new gene feature selection strategy benchmark.

## 4. Conclusions and Future Work

This paper proposed a novel structure for selecting the most informative genes from publicly available microarray data. Cancer is a progressive disease in the human body that occurs due to abnormal cell growth. If the cells reach a malignant stage, they are transformed into cancer. Abnormal cell growth occurs due to the unnatural behavior of genes. Nevertheless, all genes are not responsible for eccentric cell behavior. Therefore, it is imperative to identify these genes that behave unnaturally, and treat them using gene therapy to cure the respective disease.

The proposed PPIGCF is a three-layer gene filtration technique that can be used to fulfill the above objectives. The genes that do not participate in protein–protein interaction are eliminated in the first step. Furthermore, these genes do not create any proteins and do not participate in cellular division. In the second step, PCC is computed for every gene, and if a negative value is found, the genes are eliminated from the data. Additionally, the NSCC for every gene is calculated. If it is higher than Pearson’s correlation coefficient or tends toward one, this gene is treated as a bad gene and eliminated. In the final step, the IC value of each selected gene and the IC threshold value from gene ontology analysis are computed. If the IC threshold value is more significant than the IC value of a gene, this gene is eliminated. The satisfactory performance of PPIGCF compared to the methods used in other recent papers shows that the identified genes are most significant for the experimental microarray data.

Nevertheless, some areas require further progress. By using this technique, mutant genes can be identified. However, this technique cannot conclude the step-by-step mutation of a gene due to tumor progression [70,71,72,73]. Each step of the progression must be preserved, the gene expression data should be collected, and an analysis should be performed. However, this would take quite a long time, and the cost of this method is also higher. Nevertheless, if it is possible to optimize this method, then there is a chance to stop mutation before the malignant stage, and the chances of cancer occurring can be reduced.

## Figures and Tables

**Table 1:** Summary of microarray datasets.

Dataset	No. of Genes	Class Name (Class1/Class2)	No. of Samples (Class1/Class2)
Leukemia	7129	AML/ALL	25/47
Colon	2000	Positive/Negative	22/40
DLBCL	7129	FL/DLBCL	19/58
Lung	12,533	Mesothelioma/ADCA	31/150
Prostate	12,600	Normal/Tumor	59/77

**Table 2:** Obtained IC thresholds with GOA.

GOA	ECC	RES	Seq	Pfam
ABP	0.5949	0.9762	0.5765	0.6726
AMF	0.7293	0.9076	0.4679	0.4648
ACC	0.6549	0.9371	0.4960	0.3741
MBP	0.6443	0.9605	0.5703	0.6502
MMF	0.6754	0.8966	0.4171	0.4311
MCC	0.6875	0.9110	0.4725	0.3512

**Table 3:** Classification results of PPIGCF.

Dataset	Leukemia	Colon Cancer	DLBCL	Lung Cancer	Prostate Cancer
No. of genes (original)	7129	2000	7129	12533	12600
No. of genes (*PPIGCF*)	1849	1230	1898	2926	2737
Accuracy (%) KNN (original)	82.78	82.15	81.41	89.31	85.36
Accuracy (%) KNN (*PPIGCF*)	96.56	97.3	96.05	96.88	97.47
Accuracy (%) RF (original)	87.05	84.9	82.75	86.62	84.68
Accuracy (%) RF (*PPIGCF*)	98.14	98.99	98.84	98.5	98.67
Accuracy (%) SVM (original)	84.61	84.14	83.66	87.54	85.6
Accuracy (%) SVM (*PPIGCF*)	99.84	99.59	99.34	99.11	99.22
Accuracy (%) Naïve Bayes (original)	84.82	83.71	82.6	87.83	85.21
Accuracy (%) Naïve Bayes (*PPIGCF*)	95.18	93.62	94.07	93.49	94.78

**Table 4:** KEGG pathways mapped to leukemia genes obtained from FRD.

KEGG Pathway Name	Fold Enrichment Score	Significance
Arachidonic Acid Metabolism	65.9	Arachidonic acid and its derivatives are directly linked to the immune system and inflammation. Due to their nature, the arachidonic acid metabolism function can be held responsible for the prognosis of frequently occurring diseases [55].
Pancreatic Secretion	26.6	Abnormality in secretory processes may occur due to the malignant nature of the cells [56].

**Table 5:** KEGG pathways mapped to colon cancer genes obtained from FRD.

KEGG Pathway Name	Fold Enrichment Score	Significance
Small-Cell Lung Cancer	29.15	SCLC has a high proliferation rate. It has a strong predilection and early metastasis. Its mapping, therefore, is significant for the general diagnosis of cancer [57].
Neuroactive Ligand–Receptor Interaction	11.50	Ligand–receptor interactions are significant protein–protein interactions that play a major role in influencing biological processes, such as metabolism, neurotransmission, and cellular signal transduction pathways. Thus, this pathway network can play a huge role in cancer prognosis and detection [58].

**Table 6:** KEGG pathways mapped to lung cancer genes obtained from FRD.

KEGG Pathway Name	Fold Enrichment Score	Significance
Complement and Coagulation Cascade	31.93	The complement system serves as the main component of the immunity system, whereas the coagulation system is the pillar supporting hemostasis. Interaction between these two cascades is often proposed but has not yet been established via clinical trials [59].
Carbon Metabolism	23.32	Altered carbon metabolism plays a critical role in rapid and unregulated proliferation. One-carbon metabolism plays a significant role in DNA synthesis. Therefore, its role in cancer prognosis is essential [60].
Fluid Shear Stress and Atherosclerosis	19.43	Shear stress caused by liquid flow plays a significant role in cancer development. It affects tumor progression by actively participating in tumor cell proliferation, apoptosis, invasion, and metastasis [61].

**Table 7:** KEGG pathways mapped to DLBCL cancer genes obtained from FRD.

KEGG Pathway Name	Fold Enrichment Score	Significance
Pentose and Glucuronate Interconversions	67.05	This pathway forms the basis of other critical pathways, such as the carbohydrate metabolic pathway. It is involved in the interconversion of monosaccharide pentose and glucuronate, the salts or esters of glucuronic acid. This pathway includes 28 different members, according to the KEGG database. The pentose and glucuronate interconversion pathways play a significant role in many biosynthetic processes. It can be said that aberrant pentose and glucuronate interconversions can lead to various diseases, such as familial tumoral calcinosis [62].
Aldosterone-regulated Sodium Reabsorption	61.61	The epithelial sodium channelplays a significant role in cancer cell proliferation [63].
N-Glycan Biosynthesis	45.59	Glycosylation induces significant functional changes in various glycoproteins, including cell surface receptors, adhesion molecules, etc. These changes confer unique characteristics and phenotypes associated with cancer cells [64].
P-53 Signaling Pathway	31.23	Activated by various stresses, genotoxic damage, etc., the P-53 signaling pathway can arrest the growth of cancer cells. Therefore, it is suitable for cancer detection and feature extraction analysis from biological datasets [65].

**Table 8:** KEGG pathways mapped to prostate cancer genes obtained from FRD.

KEGG Pathway Name	Fold Enrichment Score	Significance
Biosynthesis of Cofactors	24.50	The Biosynthesis of cofactors can disrupt natural cell proliferation by rewriting cellular signaling and reprogramming the metabolic pathways [66].
Tryptophan Metabolism	60.30	This pathway is responsible for the aging process. It produces metabolites responsible for controlling inflammation, regulating energy homeostasis, and modulating behavior [67]. The tryptophan pathway promotes the intrinsic malignant properties of tumor cells, and at the same time, also restricts anti-tumor immunity. Thus, it has been targeted for drug design to produce efficient defense against tumor cell replication [68].

**Table 9:** Performance analysis of PPIGCF based on the number of genes and classification performance (CP).

Dataset	Saeid et al. [28]		Bhui et al. [29]		Raj et al. [30]		PPIGCF	
	No. of Genes	CP (%)	No. of Genes	CP (%)	No. of Genes	CP (%)	No. of Genes	CP (%)
Leukemia	2132	88.06	2134	89.66	2341	94.82	1849	99.84
Colon	1812	83.92	1632	86.62	1530	93.71	1230	99.59
DLBCL	4312	84.77	4565	88.19	4762	92.60	1898	99.34
Lung	7856	89.52	7650	90.12	8922	97.83	2926	99.11
Prostate	7769	82.98	7856	86.79	7650	95.21	2737	99.22

**Table 10:** Computing performance of PPIGCF analysis.

Dataset	PCA (s)	ICA (s)	L1-Regularized Filter (s)	Saeid et al. [28] (s)	Bhui et al. [29] (s)	Raj et al. [30] (s)	PPIGCF (s)
Leukemia	433	531	**140**	182	333	233	152
Colon	361	264	107	179	161	152	**99**
DLBCL	441	242	**132**	189	341	229	145
Lung	756	566	**234**	287	556	453	279
Prostate	648	439	227	238	448	341	**212**

## Data Availability

All data supporting reported results can be found at https://github.com/ayanban011/HandsonML/tree/main/bioinformatics (accessed on 3 May 2023).

## Supplementary Materials

The following supporting information can be downloaded at: https://www.mdpi.com/article/10.3390/genes14051063/s1, File S1: Table S1: Gene classification based on GO similarity; Table S2: Gene reduction based on PPI network; Table S3: Elimination of genes through PCC and NSCC; Table S4: Elimination of genes with respect to IC value.
