# Supplementary material for: PPIGCF: A Protein–Protein Interaction-Based Gene Correlation Filter for Optimal Gene Selection
Source: Genes (Basel). 2023 May 10;14(5):1063. doi: 10.3390/genes14051063 (PMC10218330; doi:10.3390/genes14051063)
Supplement: Supplementary file 1 [file genes-14-01063-s001.zip › genes-2237869-supplementary.pdf]

Article

PPIGCF: A Protein–Protein Interaction-Based Gene Correlation Filter for Optimal Gene Selection

Soumen Kumar Pati [1,†], Manan Kumar Gupta [1,†], Ayan Banerjee [2,†], Saurav Mallik [3,4,5] and Zhongming Zhao [3,6,*]

[1] Department of Bioinformatics, Maulana Abul Kalam Azad University of Technology, Haringhata 741249, West Bengal, India
[2] Department of Computer Science and Engineering, Jalpaiguri Govt. Engineering College, Jalpaiguri 735102, West Bengal, India
[3] Center for Precision Health, School of Biomedical Informatics, The University of Texas Health Science Center at Houston, Houston, TX 77030, USA; smallik@arizona.edu
[4] Department of Environmental Health, Harvard T H Chan School of Public Health, Boston, MA 02115, USA
[5] Department of Pharmacology & Toxicology, University of Arizona, Tucson, AZ 85721, USA
[6] Human Genetics Center, School of Public Health, The University of Texas Health Science Center at Houston, Houston, TX 77030, USA
* Correspondence: zhongming.zhao@uth.tmc.edu
† These authors contributed equally to this work.

Supplementary Materials

Table S1: Gene classification based on GO similarity

GO ID	CC	Leukemia	Colon	DLBCL	Lung	Prostate
GO:0003674	Molecular function	712	223	797	1392	1400
GO:0005764	Lysosome	729	222	791	1091	1400
GO:0005783	endoplasmic reticulum	793	225	393	1398	1400
GO:0005794	Golgi apparatus	792	220	765	1456	1400
GO:0005886	plasma membrane	761	211	987	1154	1400
GO:0008150	Biological process	994	229	1567	1393	1400
GO:0016021	An integral component of the membrane	345	222	723	1241	1400
GO:0005737	Cytoplasm	1093	223	696	1397	1400
GO:0015630	Microtubule cytoskeleton	793	221	799	1345	1400

Table S2: Gene reduction based on PPI network

GO ID	Leukemia		Colon		DLBCL		Lung		Prostate	
	# genes	*IIG*	*#genes*	*IIG*	*# genes*	*IIG*	*#genes*	*IIG*	*# genes*	*IIG*
GO:0003674	712	39	223	23	797	139	1392	34	1400	186
GO:0005764	729	135	222	22	791	118	1091	86	1400	137
GO:0005783	793	61	225	25	393	91	1398	186	1400	194
GO:0005794	792	43	220	20	765	54	1456	137	1400	139
GO:0005886	761	77	211	21	987	131	1154	194	1400	118
GO:0008150	994	131	229	29	1567	103	1393	84	1400	131
GO:0016021	345	23	222	22	723	89	1241	61	1400	103
GO:0005737	1093	59	223	23	696	24	1397	7	1400	166
GO:0015630	793	97	221	21	799	166	1345	30	1400	131

Table S3: Elimination of genes through PCC and NSCC

**GO ID**	**Leukemia**			**Colon**			**DLBCL**		
	#gene	PCC	NSCC	#gene	PCC	NSCC	#gene	PCC	NSCC
GO:0003674	473	25	4	182	23	3	558	68	86
GO:0005764	434	20	55	171	22	2	573	95	38
GO:0005783	632	41	66	157	25	5	202	75	53
GO:0005794	643	19	37	200	20	2	511	78	85

GO:0005886	584	73	73	190	21	10	856	97	47
GO:0008150	763	72	79	154	29	9	364	28	50
GO:0016021	222	89	81	162	22	12	534	93	17
GO:0005737	144	84	5	197	23	3	572	10	21
GO:0015630	496	68	87	102	21	12	533	14	19

**GO ID**	**Lung**			**Prostate**		
	#gene	PCC	NSCC	#gene	PCC	NSCC
GO:0003674	358	96	74	357	186	34
GO:0005764	1105	98	12	1003	137	86
GO:0005783	212	39	40	1211	194	186
GO:0005794	1139	57	55	318	139	137
GO:0005886	960	97	6	962	118	194
GO:0008150	309	64	31	319	131	84
GO:0016021	1180	83	96	1170	103	61
GO:0005737	1139	70	51	380	166	7
GO:0015630	315	30	46	325	131	30

Table S4: Elimination of genes with respect to IC value

**GO ID**	**Leukemia**			**Colon**			**DLBCL**		
	#genes	$< \delta$	#final genes	#genes	$< \delta$	#final genes	#genes	$< \delta$	#final genes
GO:0003674	644	97	547	174	33	141	504	130	374
GO:0005764	459	9	450	176	32	144	540	63	477
GO:0005783	627	138	489	170	35	135	164	4	160
GO:0005794	687	159	528	178	30	148	548	139	409
GO:0005886	538	121	417	159	31	128	712	145	567
GO:0008150	712	45	667	162	39	123	386	96	190

GO:0016021	152	7	145	166	32	134	534	71	463
GO:0005737	955	126	829	174	33	141	641	58	583
GO:0015630	541	85	456	167	31	136	600	182	418

**GO ID**	**Lung**			**Prostate**		
	#genes	< δ	#final genes	#gene	< δ	#final genes
GO:0003674	188	114	74	358	139	219
GO:0005764	895	194	601	1005	118	887
GO:0005783	133	56	77	212	91	121
GO:0005794	207	145	62	319	54	265
GO:0005886	857	94	763	960	131	829
GO:0008150	214	69	145	309	103	206
GO:0016021	1001	101	900	180	79	101
GO:0005737	269	100	169	390	24	366
GO:0015630	239	62	177	315	166	149

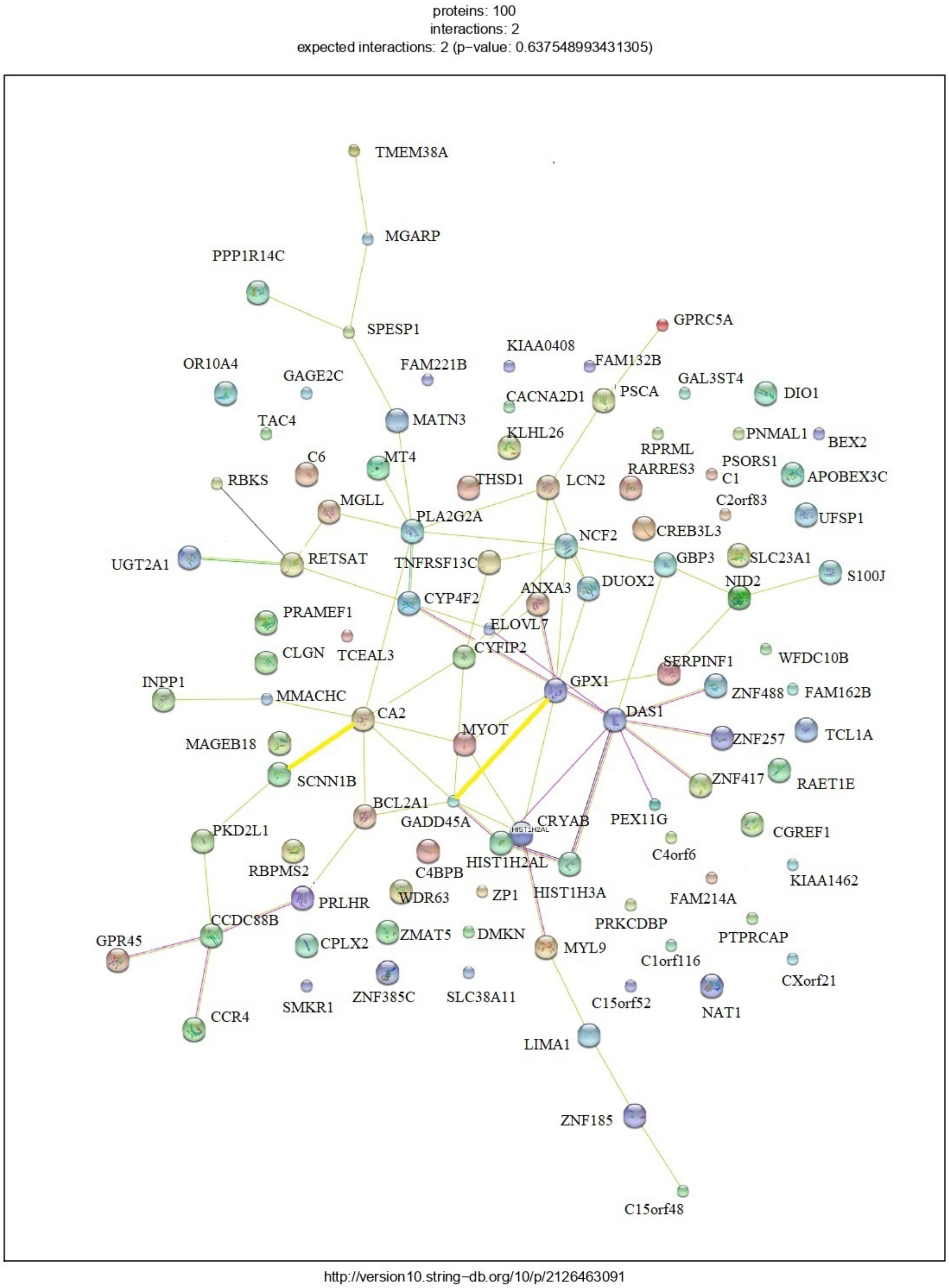
proteins: 100
interactions: 2
expected interactions: 2 (p-value: 0.637548993431305)
TMEM38A
MGARP
PPP1R14C
SPESP1
GPRC5A
KIAA0408
FAM132B
OR10A4
GAGE2C
FAM221B
PSCA
GAL3ST4
DIO1
CACNA2D1
MATN3
TAC4
KLHL26
RPRML
PNMAL1
BEX2
C6
MT4
PSORS1
C1
APOBEX3C
RBKS
THSD1
LCN2
RARRES3
MGLL
C2orf83
PLA2G2A
UFSP1
NCF2
CREB3L3
UGT2A1
RETSAT
TNFRSF13C
GBP3
SLC23A1
S100J
DUOX2
NID2
ANXA3
CYP4F2
PRAMEF1
ELOVL7
TCEAL3
CYFIP2
WFDC10B
CLGN
SERPINF1
INPP1
GPX1
ZNF488
FAM162B
MMACHC
CA2
DAS1
MYOT
ZNF257
TCL1A
MAGEB18
SCNN1B
ZNF417
RAET1E
BCL2A1
PEX11G
GADD45A
CRYAB
PKD2L1
CGREF1
C4orf6
HIST1H2AL
RBPMS2
C4BPB
HIST1H3A
KIAA1462
FAM214A
PRLHR
WDR63
ZP1
CCDC88B
PRKCDBP
GPR45
PTPRCAP
ZMAT5
DMKN
CPLX2
MYL9
C1orf116
CXorf21
ZNF385C
SLC38A11
SMKR1
C15orf52
NAT1
CCR4
LIMA1
ZNF185
C15orf48
http://version10.string-db.org/10/p/2126463091

(a)

proteins: 100
interactions: 2
expected interactions: 1 (p-value: 0.573393582116955)

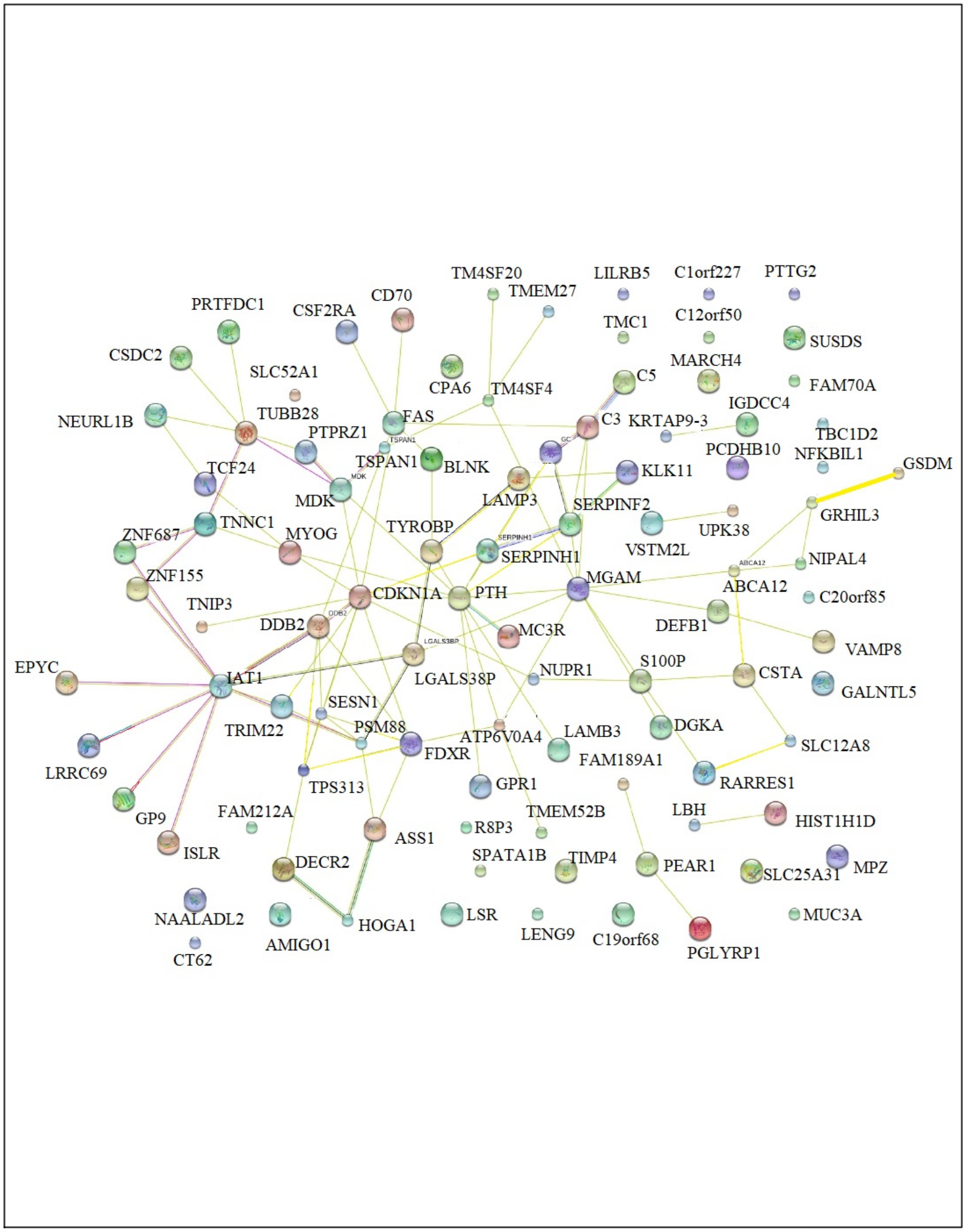

http://version10.string-db.org/10/p/1257463092

(b)

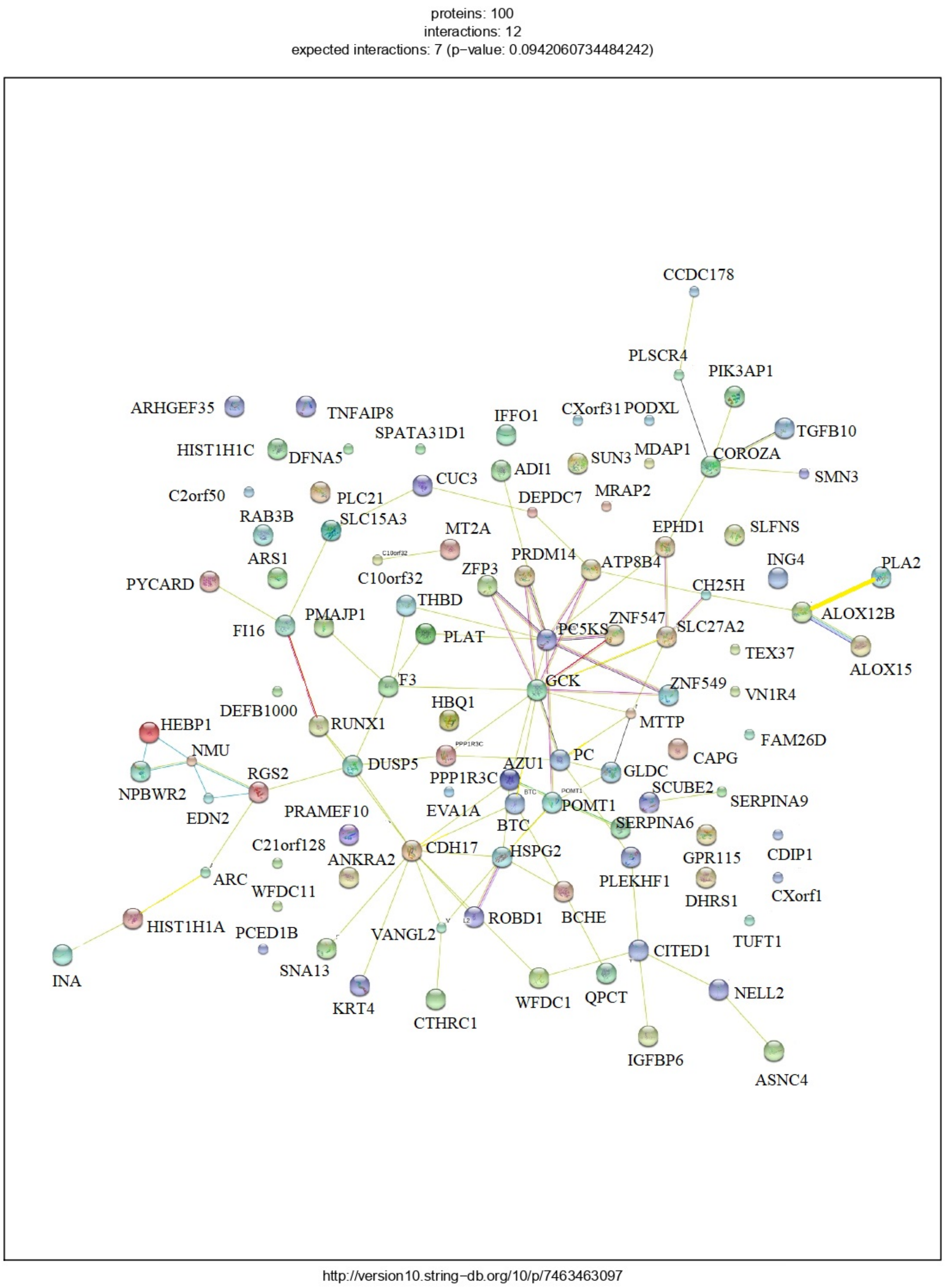
proteins: 100
interactions: 12
expected interactions: 7 (p-value: 0.0942060734484242)
CCDC178
PLSCR4
PIK3AP1
ARHGEF35
TNFAIP8
IFFO1
CXorf31
PODXL
TGFB10
HIST1H1C
DFNA5
SPATA31D1
SUN3
MDAP1
COROZA
SMN3
C2orf50
PLC21
CUC3
ADI1
DEPDC7
MRAP2
RAB3B
SLC15A3
MT2A
EPHD1
SLFNS
ARS1
C10orf32
PRDM14
ATP8B4
ING4
PLA2
PYCARD
ZFP3
CH25H
THBD
ALOX12B
FI16
PMAJP1
PC5KS
ZNF547
SLC27A2
PLAT
TEX37
ALOX15
F3
GCK
ZNF549
VN1R4
DEFB1000
HBQ1
MTTP
HEBP1
RUNX1
FAM26D
NMU
CAPG
RGS2
DUSP5
PPP1R3C
AZU1
PC
GLDC
NPBWR2
SCUBE2
SERPINA9
EDN2
EVA1A
POMT1
PRAMEF10
BTC
SERPINA6
C21orf128
CDH17
HSPG2
GPR115
CDIP1
ANKRA2
PLEKHF1
ARC
WFDC11
DHRS1
CXorf1
HIST1H1A
PCED1B
VANGL2
ROBD1
BCHE
TUFT1
INA
SNA13
CITED1
KRT4
NELL2
CTHRC1
WFDC1
QPCT
IGFBP6
ASNC4
http://version10.string-db.org/10/p/7463463097

(c)

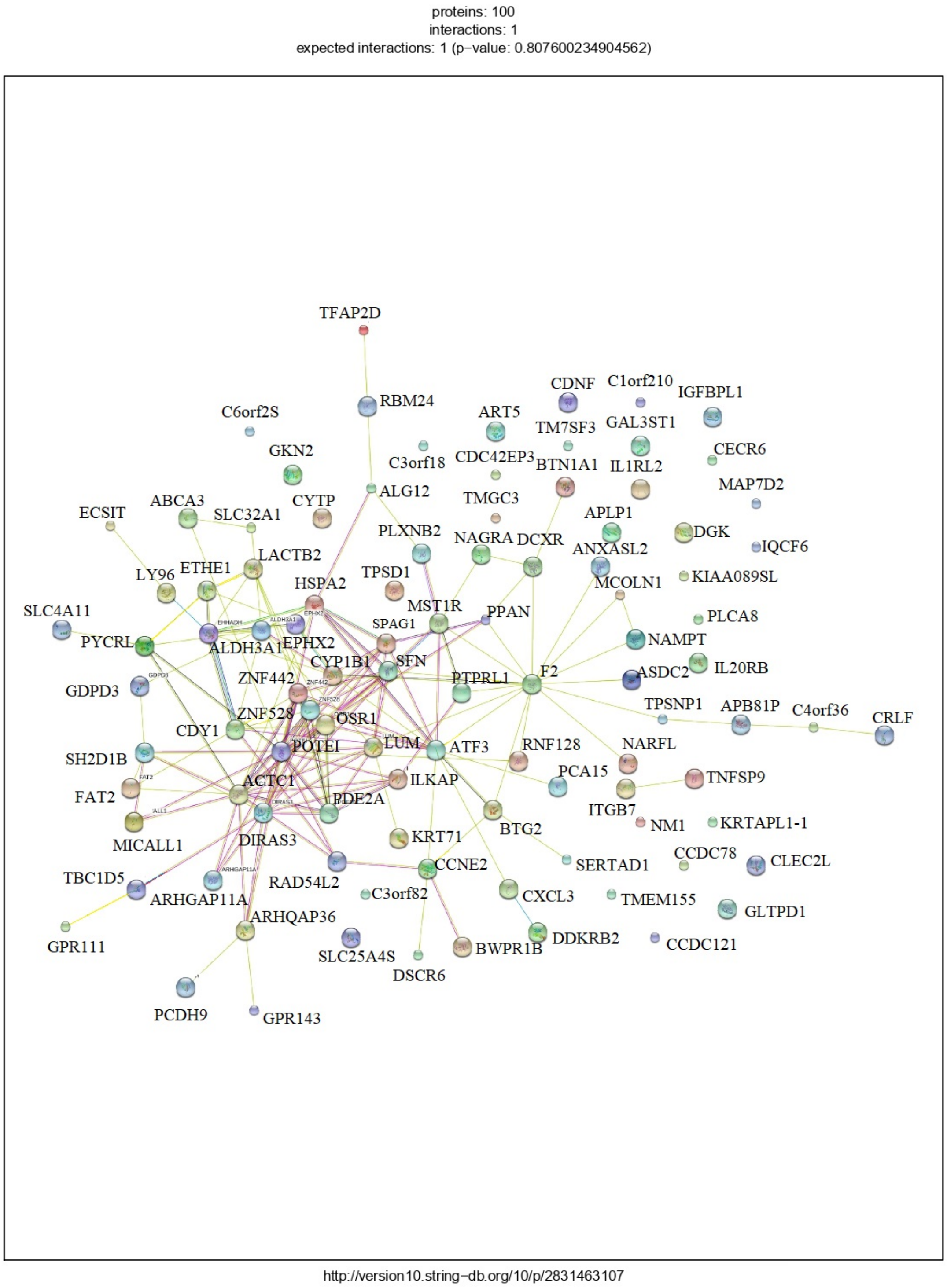
proteins: 100
interactions: 1
expected interactions: 1 (p−value: 0.807600234904562)
TFAP2D
CDNF
C1orf210
IGFBPL1
RBM24
ART5
C6orf2S
TM7SF3
GAL3ST1
GKN2
C3orf18
CDC42EP3
BTN1A1
IL1RL2
CECR6
ALG12
MAP7D2
ECSIT
ABCA3
SLC32A1
CYTP
TMGC3
APLP1
DGK
PLXNB2
NAGRA
DCXR
ANXASL2
IQCF6
LACTB2
LY96
ETHE1
TPSD1
MCOLN1
KIAA089SL
HSPA2
SLC4A11
MST1R
PPAN
PLCA8
SPAG1
PYCRL
ALDH3A1
EPHX2
NAMPT
CYP1B1
SFN
IL20RB
ZNF442
ASDC2
PTPRL1
F2
GDPD3
ZNF528
TPSNP1
APB81P
C4orf36
CRLF
OSR1
CDY1
SH2D1B
POTEI
LUM
ATF3
RNF128
NARFL
PCA15
ILKAP
ACTC1
TNFSP9
FAT2
PDE2A
ITGB7
NM1
KRTAPL1-1
BTG2
MICALL1
DIRAS3
KRT71
SERTAD1
CCDC78
CLEC2L
CCNE2
TBC1D5
RAD54L2
ARHGAP11A
C3orf82
CXCL3
TMEM155
GLTPD1
ARHQAP36
GPR111
DDKRB2
SLC25A4S
BWPR1B
CCDC121
DSCR6
PCDH9
GPR143
http://version10.string-db.org/10/p/2831463107

(d)

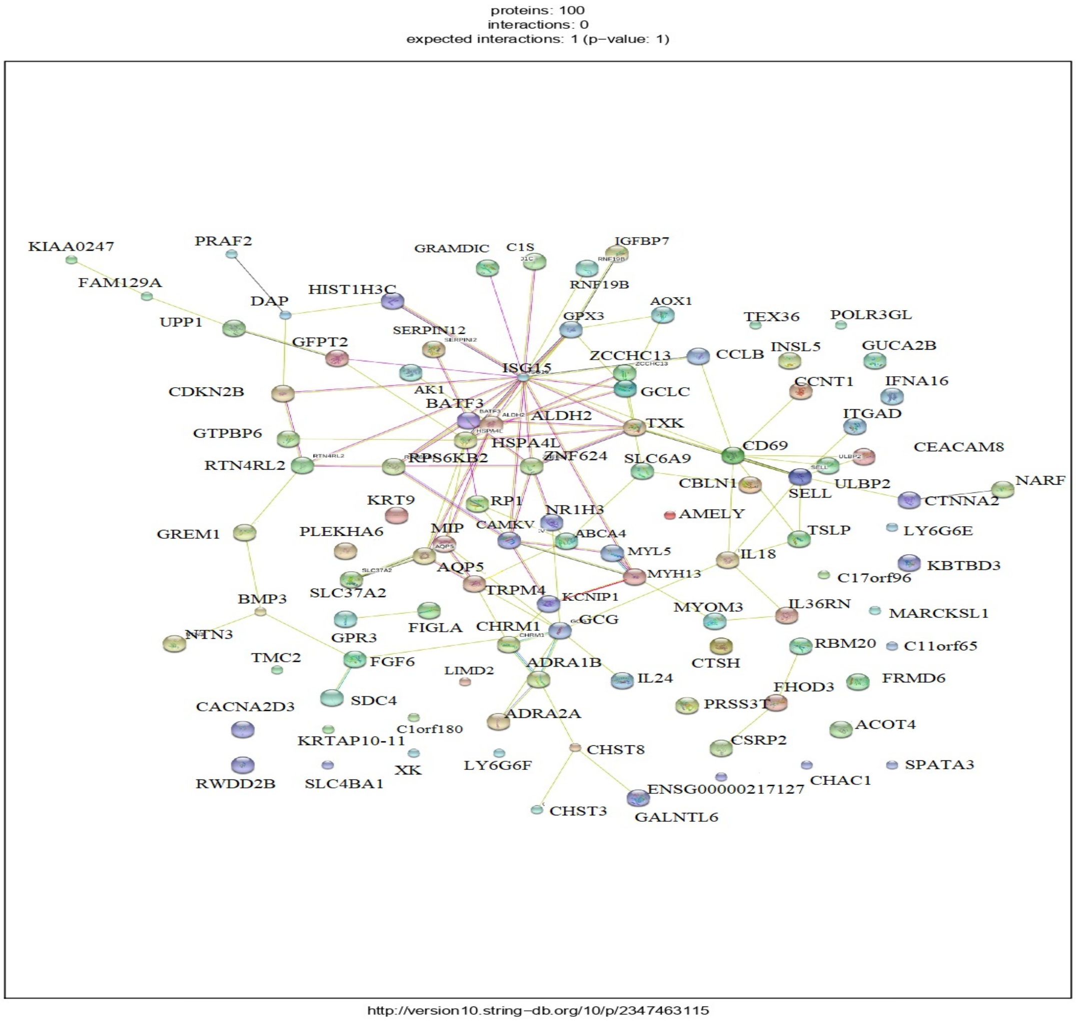

(e)

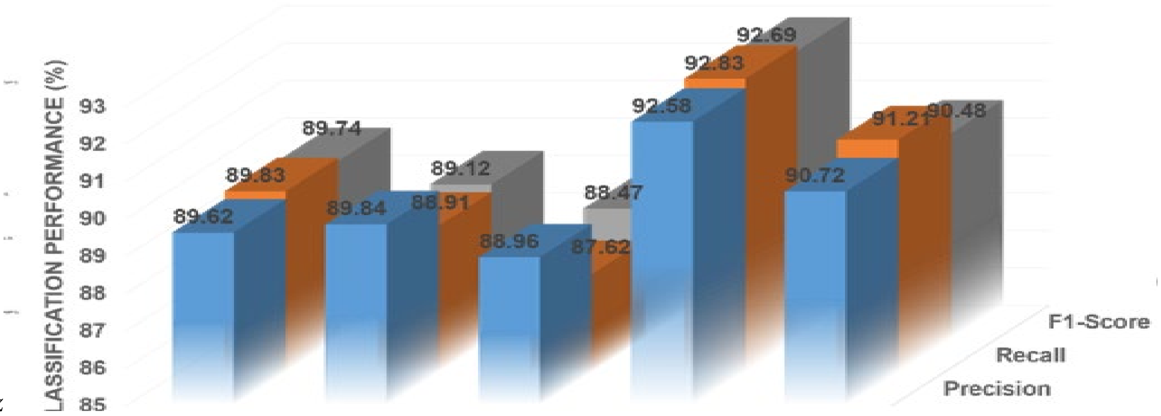

(f)

Figure S1. Protein-Protein Interactions network of GO:0003674 group (a) Leukemia, (b) Colon, (c) Lung (d) DLBCL, (e) Prostate & (f) classification performance of RD1.
